# Immunometabolic and immune regulatory functions of capsaicin in cancer: mechanistic insights and emerging perspectives

**DOI:** 10.3389/fimmu.2026.1806015

**Published:** 2026-05-29

**Authors:** Kun Xie, Zhihong Qi, Zhuo Chen, Rui Zhang, Liang Liu, Weini Li, Duoyao Cao, Daxing Xie, Jie Shen

**Affiliations:** 1 Department of GI Surgery, Tongji Hospital, Tongji Medical College, Huazhong University of Science and Technology, Wuhan, China; 2 Molecular Medicine Center, Tongji Hospital, Tongji Medical College, Huazhong University of Science and Technology, Wuhan, China; 3 Department of Biomedical Science, Cedars-Sinai Medical Center, Cedars-Sinai Cancer Institute, Los Angeles, CA, United States; 4 International Center for Aging and Cancer, Key Laboratory of Reproductive Health Diseases Research and Translation of Ministry of Education, The First Affiliated Hospital, Hainan Medical University, Haikou, China

**Keywords:** cancer therapy, capsaicin, immune cells, immune metabolism, tumor immune microenvironment

## Abstract

Capsaicin, a natural bioactive alkaloid derived from chili peppers, has garnered increasing interest for its broad spectrum of pharmacological activities. Beyond its well-recognized analgesic, anti-inflammatory, and metabolic regulatory properties, accumulating evidence underscores its emerging roles in tumor suppression and immune modulation. Recent studies demonstrate that capsaicin profoundly influences the function and metabolism of diverse immune cell populations—including T cells, natural killer cells, macrophages, and dendritic cells—thereby enhancing antitumor immunity and immune surveillance. Building upon these findings, recent studies support combinatorial strategies that integrate capsaicin with conventional anticancer therapies to improve chemosensitivity and therapeutic efficacy. This review summarizes the latest advances in understanding how capsaicin regulates immunometabolism and remodels the tumor immune microenvironment, with an emphasis on the molecular mechanisms underlying its antitumor activity and potential implications for future therapeutic development.

## Introduction

Capsaicin, the principal pungent alkaloid responsible for the pungency of Capsicum species, is widely recognized for its distinctive spicy flavor. Beyond its traditional use as a food additive, capsaicin has recently gained considerable attention owing to its broad range of pharmacological activities, including analgesic, anti-inflammatory, antioxidant, metabolic regulatory, and anticancer effects. Experimental studies have demonstrated that capsaicin not only acts directly on tumor cells—by inducing apoptosis, arresting the cell cycle, inhibiting proliferation, and suppressing metastasis—but also exerts indirect antitumor effects through modulation of the host immune system (1).

The tumor immune microenvironment (TIME) is a central determinant of tumor initiation, progression, and therapeutic resistance. Immune cells such as T cells, natural killer (NK) cells, dendritic cells (DCs) and myeloid cells are critical mediators of immune surveillance and immune escape in cancer (2, 3). In recent years, diverse immunotherapeutic strategies—including immune checkpoint blockade, adoptive cell transfer, and tumor vaccination—have revolutionized cancer treatment and underscored the pivotal role of the immune system in tumor control (4). However, the pronounced heterogeneity and immunosuppressive features of the TIME often limit the efficacy and durability of current immune-based therapies. These challenges highlight the urgent need for novel immunomodulatory agents capable of enhancing existing therapeutic modalities.

Emerging evidence indicates that capsaicin can reprogram the functional state and polarization of immune cells within the TIME, thereby alleviating immunosuppressive conditions. For example, capsaicin has been shown to inhibit the polarization of tumor-associated macrophages toward an M2-like immunosuppressive phenotype (5) and to enhance antigen-presenting capacity in dendritic cells, ultimately promoting the activation of cytotoxic T lymphocytes and NK cells. Collectively, these findings suggest that capsaicin, as a natural small-molecule compound, holds promise as an innovative modulator of the TIME capable of reinforcing antitumor immunity. This review focuses on the emerging role of capsaicin in immune-metabolic regulation within the TIME. In addition, we highlight critical knowledge gaps, including context-dependent immune effects, dose-dependent functional divergence, and the limited translational validation of these mechanisms.

## Capsaicin and its receptor TRPV1

Capsaicin is a lipophilic vanillylamide with the molecular formula C_18_H_27_NO_3_. It dissolves readily in most organic solvents but is poorly soluble in water (6), which may limit its systemic availability. Its oral bioavailability is relatively low due to extensive hepatic metabolism by cytochrome P450 enzymes; however, its slower cutaneous metabolism renders it well suited for topical analgesic formulations (7).

Early studies demonstrated that Capsaicin selectively activates nociceptive fibers in sensory neurons, evoking sensations of burning, stinging, and warmth (8). This discovery led to the identification of a specific molecular target—the Transient Receptor Potential Vanilloid 1 (TRPV1), also known as the vanilloid receptor 1 (VR1). TRPV1 is a nonselective, ligand-gated cation channel expressed in the plasma membrane and endoplasmic reticulum of primary sensory neurons. It is activated by heat (≥43 °C), acidic conditions, and a variety of chemical ligands (9). Upon capsaicin binding, TRPV1 undergoes conformational rearrangement that opens the channel pore, allowing the influx of Ca²^+^ and Na^+^ ions. The resultant depolarization generates action potentials that propagate along nociceptive fibers, eliciting the characteristic burning sensation.

Prolonged or high-dose capsaicin exposure can induce TRPV1 receptor desensitization, thereby diminishing responsiveness to subsequent stimuli. This adaptive process involves multiple mechanisms, including intracellular Ca²^+^ overload, regulation of calcium-dependent protein kinases (such as protein kinase C) and calmodulin, and depletion of neurotransmitters such as substance P and calcitonin gene related peptide (CGRP) (10–12). In parallel, vesicular release at sensory nerve terminals may be transiently impaired, leading to mild axonal degeneration and decreased nociceptive transmission.

Beyond pain perception, TRPV1 signaling participates in diverse physiological processes within both the central and peripheral systems, including inflammation, energy metabolism, cardiovascular regulation, and tumorigenesis (8). In some contexts, TRPV1 activation may promote the release of endogenous peptides and vasoactive mediators, thereby affecting local blood flow and inflammatory responses. Importantly, the broad biological functions of TRPV1 provide a mechanistic basis for understanding how capsaicin may influence not only sensory signaling but also tumor-related immune and metabolic processes discussed in the following sections.

## Capsaicin in cancer biology

Accumulating evidence suggests that capsaicin can induce apoptotic cell death in several tumor models (1). Capsaicin has been shown to induce apoptosis in diverse tumor cell types and to suppress aberrant nuclear factor kappa B (NF-κB) and mitogen-activated protein kinase (MAPK) signaling pathways (13), thereby contributing to its antitumor potential. In colorectal cancer cells, capsaicin activates the TRPV1, leading to Ca²^+^ influx, calcineurin activation, dephosphorylation, and nuclear translocation of nuclear factor of activated T cells 2 (NFAT2), followed by p53 activation and apoptosis (14). Similarly, in breast cancer cells, capsaicin modulates the factor that binds to the inducer of short transcripts of HIV-1 (FBI-1), thereby affecting the nuclear factor kappa B (NF-κB) pathway and its downstream effectors, suppressing proliferation and enhancing apoptotic signaling (15). In addition, capsaicin promotes Ca²^+^ influx through TRPV1 channels, leading to mitochondrial membrane depolarization, disruption of the electron transport chain, and accumulation of reactive oxygen species (ROS), which may collectively contribute to oxidative stress and cell death (16). Together, these findings suggest that calcium dysregulation, mitochondrial dysfunction, and stress-related signaling are common mechanisms underlying capsaicin-induced apoptosis in cancer cells.

Dysregulation of the cell cycle represents another major mechanism driving uncontrolled tumor growth. Several studies suggest that capsaicin can interfere with several cell-cycle checkpoints, thereby restraining tumor cell proliferation. Islam et al. (17) reported that in bladder cancer cells, capsaicin binds to and promotes the degradation of tumor-associated NADH oxidase (tNOX), resulting in inhibition of the NAD-dependent deacetylase SIRT1. This process increases the acetylation of MYC proto-oncogene, bHLH transcription factor (c-Myc) and p53, leading to G_1_-phase arrest. In colorectal cancer, capsaicin disrupts the interaction between p53 and mouse double minute 2 homolog (MDM2), stabilizing p53 and inducing G_0_/G_1_-phase arrest (18). In breast cancer models, it downregulates cyclin-dependent kinase 8 (CDK8) expression, resulting in G_2_/M-phase blockade (19). These findings indicate that capsaicin-mediated cell-cycle arrest may involve multiple regulatory nodes, particularly those associated with p53 signaling and cell-cycle checkpoint control.

The role of capsaicin in autophagy regulation appears to be more complex and context-dependent. Autophagy can either promote tumor cell survival under stress or facilitate cell death when excessively activated (20). For example, Yu-Tsai Lin et al. (21) observed that in nasopharyngeal carcinoma cells, capsaicin treatment increased LC3-II and Atg5 levels while decreasing p62 and Fap-1 expression, concomitant with elevated caspase-3 activity—indicative of concurrent activation of autophagy and apoptosis. In contrast, other studies have suggested that capsaicin-induced autophagy may act as a cytoprotective mechanism that enables tumor adaptation to stress (20).

Capsaicin has also been reported to modulate several key oncogenic signaling pathways, including NF-κB, phosphoinositide 3-kinase/protein kinase B (PI3K/Akt), AMP-activated protein kinase (AMPK), and p38 mitogen-activated protein kinase (p38 MAPK) (13). However, these pathways should not be viewed as isolated events, as they collectively influence key malignant phenotypes. For example, NF-κB signaling, which is closely associated with inflammation and cell survival, has been shown to be suppressed by capsaicin through downregulation of the FBI-1 protein, thereby promoting apoptosis in breast cancer cells (15). Likewise, inhibition of the PI3K/Akt pathway by capsaicin decreases the phosphorylation of PI3K and AKT and is associated with reduced tumor cell proliferation (19). In thyroid cancer cells, capsaicin downregulates matrix metalloproteinase-2 (MMP2) and matrix metalloproteinase-9 (MMP9) expression through TRPV1-dependent mechanisms, which may partially reverse epithelial–mesenchymal transition and reducing invasive potential (22). In addition, recent research has shown that capsaicin downregulates key DNA damage repair proteins, including poly (ADP-ribose) polymerase 1 (PARP1), DNA ligase 1 (LIG1), and flap endonuclease 1 (FEN1), thereby inhibiting gastric cancer cell proliferation and migration (23).

## Capsaicin in immune regulation and immunometabolism crosstalk

### Capsaicin in immune regulation

Recent studies indicate that capsaicin modulates both innate and adaptive immune responses (summarized in **Table 1**). A recent report demonstrated that in an LPS-induced inflammatory model using mouse peritoneal macrophages, capsaicin enhanced macrophage phagocytosis while suppressing the mRNA and protein expression of inflammatory mediators, including interleukin-6 (IL-6), tumor necrosis factor-α (TNF-α), and nitric oxide. This effect was accompanied by reduced NF-κB p65 phosphorylation, suggesting a potential anti-inflammatory mechanism (24). Other studies have shown that capsaicin may promote the polarization of obesity-associated inflammatory macrophages toward an M2-like, anti-inflammatory phenotype, reducing their responsiveness to lipopolysaccharide. Moreover, pretreatment with capsaicin prior to adenosine triphosphate (ATP)-induced NOD-like receptor family pyrin domain containing 3 (NLRP3) inflammasome activation conferred anti-inflammatory effects by inhibiting inflammasome assembly and decreasing interleukin-1β (IL-1β) release in peripheral blood mononuclear cells (25).

**Table 1:** Effects of capsaicin on different immune cells.

Cell type	Model	Dose/context	TRPV1 dependence	Effect	Direction	Reference
Macrophages	*In vitro* murine peritoneal macrophages	LPS 1 μg/mL + capsaicin 25–100 μg/mL for 24 h	Not determined	Enhanced phagocytosis and reduced IL-6, TNF-α, and NO/iNOS production	Biphasic (context-dependent)	(24)
	*In vitro* murine obesity-associated inflammatory macrophages	Capsaicin 25–50 μM for 24 h	Not determined	Promoted M2-like polarization and inhibited inflammasome activation/IL-1β release	Decreased (immunosuppressive)	(25)
Dendritic cells	*In vitro* murine bone marrow-derived dendritic cells	Capsaicin (5-100μM) for 16 h	TRPV1-dependent	Promoted dendritic-cell maturation and upregulated antigen-presenting molecules	Increased (pro-immune)	(26)
	*In vivo* murine pancreatic lymph nodes	Oral capsaicin gavage (10μg per mice)	Not determined	Increased IL-27-producing dendritic-cell population	Biphasic (context-dependent)	(27)
Neutrophils	*In vivo* rat pleurisy model and murine peritonitis models	Pretreat capsaicin-containing *C. baccatum* juice (0.25–2.0 g/kg)	Not determined	Inhibited migration and reduced inflammatory responses	Decreased (immunosuppressive)	(28)
	*In vivo* human nasal challenge model in allergic rhinitis	Intranasal capsaicin spray (200μM)	Not determined	Dose-dependent increase in neutrophil recruitment	Increased (pro-immune)	(29)
NK cells	*In vitro* human NK cells	High concentrations of capsaicin (50–100 μM) for 1h	TRPV1-independent	Suppressed cytotoxic degranulation and reduced cytokine secretion	Decreased (immunosuppressive)	(30)
T cells	*In vitro* rat thymocytes	Capsaicin 5, 10, or 25 μM for 8 h	TRPV1-dependent	Induced apoptotic cell death	Decreased (immunosuppressive)	(31)
	*In vitro* naïve CD4^+^ T-cell differentiation model	Capsaicin 1-3μg/ml for 6 h	TRPV1-dependent	Promoted Th2 differentiation and reduced Th1 polarization	Decreased (immunosuppressive)	(32)
	*In vivo* intestinal neuroimmune model	Directly activate Trpv1^+^ nociceptive neurons by clozapine N-oxide	TRPV1-dependent	Reduced RORγ^+^ regulatory T-cell abundance	Increased (pro-immune)	(33)

Dendritic cells (DCs), the “sentinels” of the immune system, are primarily responsible for antigen capture, processing, and presentation to T lymphocytes. Evidence indicates that capsaicin can directly stimulate activated DCs through vanilloid receptor 1 binding, promoting their maturation by upregulating antigen-presenting and co-stimulatory molecules (26, 34). Furthermore, Nandini et al. (27) reported that dietary capsaicin suppressed the proliferation and activation of self-reactive T cells in mice while increasing IL-27-producing DCs and IL-10-secreting CD4^+^/FoxP3^-^ Tr1 cells in pancreatic lymph nodes, suggesting that capsaicin may shape DC-mediated immune tolerance and T-cell differentiation.

Neutrophils, as key effector cells of acute inflammation, play a vital role in pathogen clearance. Research on capsaicin’s effects on neutrophils suggests that it modulates neutrophil migration and inflammatory responses. In a murine pleurisy model, capsaicin treatment significantly inhibited neutrophil infiltration and reduced levels of TNF-α and IL-1β in pleural exudates (28). These effects may reflect a nonspecific inflammatory modulation mediated by sensory nerve activation, although the molecular basis remains incompletely understood (29).

Natural killer (NK) cells, essential components of innate immunity, serve as first-line defenders against tumors and viral infections (35). Several studies suggest that capsaicin can suppress NK-cell function under certain conditions. In an *in vitro* model using K562 or 221 target cancer cells, capsaicin inhibited NK cell cytotoxic degranulation and cytokine secretion in a concentration-dependent manner, with modest effects at 10–20 μM and pronounced suppression at 50–100 μM following short-term (2 h) exposure. Although NK cells express functional TRPV1, the inhibitory effect at higher concentrations appeared to be largely TRPV1-independent and was associated with defective Ca²^+^ mobilization during NK-cell activation (30). Consistently, *in vivo* evidence from rat models shows that subcutaneous administration of capsaicin (50 mg/kg) reduced NK cell–mediated and antibody-dependent cellular cytotoxicity in both spleen and peripheral blood 15 days after treatment (36).

In recent years, capsaicin’s regulatory effects on the adaptive immune system have gained increasing attention. Through the TRPV1 signaling pathway, capsaicin may influence immune cell metabolism, cytokine secretion, and gene expression, thereby modulating T-cell function (37). T cells are central to adaptive immunity, with their subsets, including T helper 1 (Th1), T helper 2 (Th2), and regulatory T (Treg) cells, playing distinct roles in immune homeostasis. Studies have shown that TRPV1 is expressed on T-cell membranes and mediates Ca²^+^ influx during CD4^+^ T-cell activation, suggesting that TRPV1 may facilitate T-cell activation (38, 39). Prolonged exposure to high concentrations of capsaicin, however, can induce apoptosis in human peripheral blood T cells via TRPV1-dependent Ca²^+^ influx (31). Interestingly, temperature elevation during fever promotes Th2 differentiation while reducing Th1 polarization of naïve CD4^+^ T cells through a TRPV1-mediated, Notch-dependent mechanism (32). Additionally, neuronal activation of intestinal TRPV1^+^ fibers has been reported to reduce RORγ^+^ regulatory T cells (Tregs), further illustrating the intricate crosstalk between the nervous and immune systems (33).

Taken together, these findings indicate that the immunological effects of capsaicin are highly context-dependent and may vary according to immune cell type, dose, and experimental conditions. Rather than exerting uniformly stimulatory or suppressive effects, capsaicin appears to reshape immune responses through interconnected pathways involving calcium signaling, inflammatory mediators, and immune cell fate. These dual effects suggest that precise modulation of capsaicin exposure may be critical for harnessing its therapeutic potential, and that its immunological outcomes should be interpreted within a broader immune-regulatory context.

### Capsaicin in immunometabolic crosstalk

Capsaicin has been reported to influence several aspects of energy metabolism, including mitochondrial activity, fatty acid oxidation, and insulin signaling. These metabolic alterations may be closely coupled to immune function, as the metabolic state of immune cells directly determines their activation, differentiation, and effector responses. In this context, TRPV1 should not be viewed solely as a nociceptive receptor, but increasingly as a neuroimmune interface through which metabolic cues and immune-cell behavior may be coordinated (40).

Lipid metabolism is a critical determinant of immune regulation through multiple mechanisms, including post-translational lipid modifications of immune-related proteins, and the modulation of immune cell functions such as macrophage activation (41–43). *In vitro* experiments have demonstrated that capsaicin significantly alters lipid metabolism by reducing intracellular triglyceride accumulation (44). In animal studies, capsaicin supplementation markedly decreased triglyceride storage in mice fed either high-fat or normal-fat diets (45–47). Additional evidence indicates that capsaicin downregulates adipogenic gene expression while strongly stimulating AMP-activated protein kinase (AMPK) activity (48). Mechanistically, capsaicinoids appear to enhance the excretion of total acidic steroids by upregulating cholesterol 7α-hydroxylase activity and suppressing hepatic liver X receptor alpha (LXRα) expression (49). Recent studies have also reported that palmitoylation of TRPV family receptors may participate in regulating receptor function and peptide-mediated phagocytic activity (50, 51). These findings raise the intriguing possibility that capsaicin may indirectly influence TRPV1 function through lipid metabolic modifications such as palmitoylation, thereby linking metabolic signaling to immune regulation. This possibility is further supported by recent macrophage-focused studies. Li et al. showed that capsaicin attenuated LPS-induced proinflammatory properties of M1 macrophages in association with NRF2 activation and reduced intracellular ROS, whereas Hsu et al. suggested that TRPV1 may also enhance macrophage responsiveness to LPS by promoting interaction with the TLR4–CD14 complex (52, 53). In parallel, Ávila et al. demonstrated that capsaicin reduced systemic inflammation and macrophage-derived foam-cell formation through PPARγ- and TRPV1-related mechanisms, reinforcing the view that lipid remodeling and immune-cell functional reprogramming are tightly interconnected (54).

Emerging evidence has unveiled a novel role of the angiotensin-converting enzyme (ACE) in modulating cellular lipid metabolism and, consequently, regulating immune cell functions such as those of neutrophils and macrophages (43, 55). Targeting ACE has therefore been proposed as a promising therapeutic strategy to enhance antitumor immunity (56). Interestingly, capsaicin has been reported to influence ACE activity (57, 58). Although direct evidence remains limited, these findings suggest that capsaicin or its structural analogues could represent novel agents capable of modulating ACE-driven metabolic reprogramming in immune cells and thereby fine-tuning immune responses.

Capsaicin also participates in glucose homeostasis regulation by enhancing insulin signaling pathways. Studies indicate that capsaicin activates the Sirtuin-6–TRPV1–CGRP signaling axis, thereby upregulating glucose transporter type 4 expression and promoting glucose uptake (59). Hwang et al. (2005) demonstrated that capsaicin stimulates intracellular reactive oxygen species generation and increases the phosphorylation levels of acetyl-CoA carboxylase and AMP-activated protein kinase (p-AMPK), rapidly activating AMPK and inhibiting adipogenesis (60). Similarly, Jin et al. reported that capsaicin-induced ROS production elevates the phosphorylation of AMPK and p38 mitogen-activated protein kinase, thereby facilitating glucose uptake (61). *In vivo* experiments by Hirata et al. revealed that capsaicin ameliorates insulin resistance in experimental models by lowering blood glucose levels and increasing circulating insulin concentrations (62). Consistently, oral administration of capsaicin in wild-type mice was shown to enhance insulin secretion from isolated pancreatic tissue through TRPV1 activation and the subsequent release of neuropeptides CGRP and substance P (63). These metabolic improvements are closely intertwined with immune regulation, as enhanced insulin sensitivity and glucose uptake can reprogram immune cell metabolism (64), thereby influencing immune-cell activation, differentiation, and cytokine production.

The gut microbiota is increasingly recognized as an important hub linking metabolism and immunity, maintaining bidirectional communication with the host through neural, endocrine, and immune pathways. Dietary capsaicin has been shown to remodel the intestinal microbial community by increasing the relative abundance of *Akkermansia muciniphila* and *Bacteroides* species while reducing pro-inflammatory taxa such as *Enterobacteriaceae* and *Desulfovibrio* (65). Recent studies have further expanded this concept by showing that capsaicin can influence host lipid metabolism through coordinated changes in bile-acid profiles and gut microbiota composition. In particular, Gong et al. demonstrated that capsaicin regulates lipid metabolism through bile acid/gut microbiota interactions, while Mahalak et al., using an *in vitro* human gut microbiota model, found that repeated capsaicin exposure increased microbial diversity and altered short-chain fatty acids (SCFAs) abundance (66, 67). These microbial shifts are accompanied by elevated production of SCFAs, which act as signaling molecules that activate G-protein-coupled receptors and inhibit histone deacetylases, thereby promoting regulatory T-cell differentiation, suppressing inflammatory cytokine secretion, and reinforcing epithelial barrier integrity (68, 69). This interpretation is also consistent with the recent synthesis by Corral-Guerrero et al., who proposed that capsaicin should be understood not only as a dietary pungent compound but also as a microbiome modulator whose systemic effects are shaped by dynamic host–microbe metabolic interactions (70). Together, these findings suggest that capsaicin may influence immunometabolic homeostasis not only through direct effects on host signaling pathways but also through microbiota-derived metabolites.

Collectively, current evidence supports that capsaicin is involved in the crosstalk between metabolic and immune pathways. These metabolic and immune regulatory effects of capsaicin should not be viewed as independent processes but rather as components of an integrated immunometabolic network. Metabolic reprogramming—such as alterations in lipid utilization, glucose uptake, and mitochondrial activity—can directly shape immune cell activation, differentiation, and effector functions. In this context, capsaicin-induced modulation of pathways including AMPK signaling, ROS generation, and gut microbiota-derived metabolites may converge to influence immune cell fate decisions and functional polarization.

Conversely, changes in immune cell states can further remodel the metabolic landscape of the TIME, creating a dynamic feedback loop between metabolism and immunity. This bidirectional interplay provides a mechanistic basis for understanding how capsaicin simultaneously impacts tumor cell biology, immune responses, and metabolic homeostasis, ultimately contributing to its context-dependent effects within the TIME.

## Immunomodulatory role of capsaicin in tumor immune microenvironment

The tumor immune microenvironment is a critical determinant of tumor initiation, progression, and therapeutic response. It consists of tumor cells, stromal components, and diverse immune and signaling molecules that collectively shape tumor behavior (71). In tumor immunology, capsaicin has been reported to modulate immune cell function, influences immune checkpoint expression, and affect cytokine and chemokine networks, thereby contributing to changes in immune infiltration and tumor growth under specific experimental conditions.

Capsaicin has been reported to exert immunomodulatory effects across multiple immune cell types and may influence the TIME in a context-dependent manner. Specifically, capsaicin has been reported to promote the activation, proliferation, and cytotoxic activity of T cells in certain tumor models. Experimental studies have demonstrated that capsaicin enhances intra-tumoral infiltration of CD8^+^ T cells—the primary effector cells responsible for directly killing tumor cells—thereby accelerating tumor regression (72). Furthermore, activation of TRPV1 on CD4^+^ T cells induces Th1-type inflammatory responses (73), suggesting that capsaicin enhances T cell–mediated antitumor immunity through TRPV1-dependent signaling. Moreover, by influencing macrophage polarization, capsaicin promotes the reprogramming of tumor-associated macrophages (TAMs) from an M2-like immunosuppressive phenotype toward an M1-like proinflammatory and antitumor phenotype (5). This phenotypic switch may represent one mechanism through which influences the TIME. Capsaicin also enhances DC cell function. For example, in bladder cancer, capsaicin-induced tumor cell apoptosis triggers CD91-mediated DC activation, facilitating antigen uptake and processing and thereby promoting efficient antigen presentation (74).

Capsaicin further modulates the local immune milieu by regulating immune checkpoint molecules, cytokines, and chemokines, thereby influencing immune infiltration and antitumor activity. Notably, capsaicin exerts context-dependent effects on immune checkpoint regulation. Morelli et al. reported that capsaicin upregulated programmed death-ligand 1 (PD-L1) expression in bladder cancer, potentially promoting immune evasion, whereas in renal cancer, capsaicin downregulated PD-L1 through a TRPV1-independent pathway, restoring tumor susceptibility to immune attack (75). Such bidirectional regulation underscores that the immunological consequences of capsaicin vary across tumor types. Importantly, PD-L1 upregulation in response to capsaicin may, in certain contexts, reflect an adaptive immune resistance mechanism triggered by tumor stress, inflammatory signaling, or immune activation. In this scenario, capsaicin-induced PD-L1 expression may mark a tumor state that is more responsive to immune checkpoint blockade, thereby positioning capsaicin as a potential sensitizer or rational combination partner for PD-1/PD-L1–targeted therapies. Conversely, in contexts where capsaicin suppresses PD-L1 expression, it may directly alleviate checkpoint-mediated immune evasion and enhance antitumor immunity in a checkpoint-independent manner. Mechanistically, these divergent effects may be linked to differential regulation of signaling pathways implicated in PD-L1 expression, including NF-κB, STAT3, and stress-response pathways, although the precise determinants remain incompletely defined. Moreover, capsaicin could regulate the production of proinflammatory cytokines, such as IL-6, IL-12, and granulocyte-macrophage colony-stimulating factor, and the immunosuppressive cytokine, such as IL-10 (76). However, these therapeutic implications remain hypothesis-generating, as current evidence is derived from heterogeneous experimental systems with variable dosing, timing, and tumor models. This context-dependent duality highlights the importance of patient stratification and tumor-specific immune profiling when considering capsaicin-based combination strategies. Further mechanistic dissection and *in vivo* validation are required before translational application can be considered.

Another key mechanism underlying capsaicin’s immunomodulatory action is the induction of immunogenic cell death (ICD), whereby dying tumor cells release damage-associated molecular patterns (DAMPs), including heat shock protein 90 (HSP90) and calreticulin (77, 78). These DAMPs act as danger signals that activate DCs, enhance antigen presentation, and stimulate antigen-specific adaptive immune responses (79, 80). Thus, in addition to its direct effects on tumor-cell viability, capsaicin may contribute to antitumor immunity by enhancing the immunogenicity of dying tumor cells. Collectively, these findings suggest that capsaicin may influence the TIME through coordinated effects on immune-cell composition, checkpoint regulation, inflammatory mediators, and antigen-presentation pathways, although these effects remain context-dependent and are primarily supported by preclinical studies.

## Prospects and challenges of capsaicin in clinical cancer therapy

The antitumor effects of capsaicin were first demonstrated primarily through cellular and animal studies, encompassing a wide range of solid malignancies (1). Capsaicin also appears to show greater promise as an adjuvant to conventional chemotherapeutic agents rather than as a monotherapy. For instance, the combination of capsaicin with gemcitabine was associated with downregulation of ATP-binding cassette subfamily C member 2, deoxycytidine kinase, and thymidine kinase in GEM-resistant bladder cancer cells, resulting in stronger tumor suppression than either treatment alone (81). Similarly, co-administration of capsaicin and paclitaxel produced superior antitumor effects in a 4T1 breast cancer xenograft model, demonstrating greater tumor growth inhibition than monotherapy (82). Parallel studies have reported comparable synergistic effects of capsaicin combination therapy in colorectal, lung, and pancreatic cancers (83, 84).

Despite these promising preclinical findings, the clinical translation of capsaicin remains limited by its low bioavailability, rapid metabolism, and short biological half-life, and potential dose-dependent adverse effects, including local irritation and TRPV1-related sensory toxicity. To overcome these limitations, various drug delivery strategies have been explored, including nanoformulations, liposomal encapsulation, and polymer-based carriers, which aim to enhance stability, improve tumor targeting, and reduce off-target effects (85–89). For example, capsaicin-loaded nanoliposomes have been shown to improve formulation performance and enhance anticancer activity *in vitro*, whereas PLGA-based nanoparticles have demonstrated improved encapsulation efficiency and pro-apoptotic activity in HepG2 cells (90, 91). These nano-formulations aim to improve tumor-targeted delivery, enable controlled release, and reduce dose-limiting local irritation or systemic toxicity. In parallel, synthetic capsaicin analogues with reduced pungency have been developed, including non-pungent TRPV1 agonists such as olvanil and naturally occurring capsinoids (e.g., capsiate), which retain TRPV1 activity while minimizing nociceptive responses (92). These analogues may provide an alternative strategy to dissociate therapeutic efficacy from the sensory toxicity associated with native capsaicin. However, despite these advances, most nano-formulations and synthetic derivatives remain at the preclinical stage, and robust clinical evidence supporting their safety, pharmacokinetics, and anticancer efficacy is still lacking.

These translational challenges highlight a critical gap between preclinical efficacy and clinical applicability. Addressing these issues will require not only pharmacological optimization but also a deeper understanding of context-dependent effects and patient-specific responses, which remain insufficiently explored in current studies.

## Conclusion and perspective

**Figure 1:**
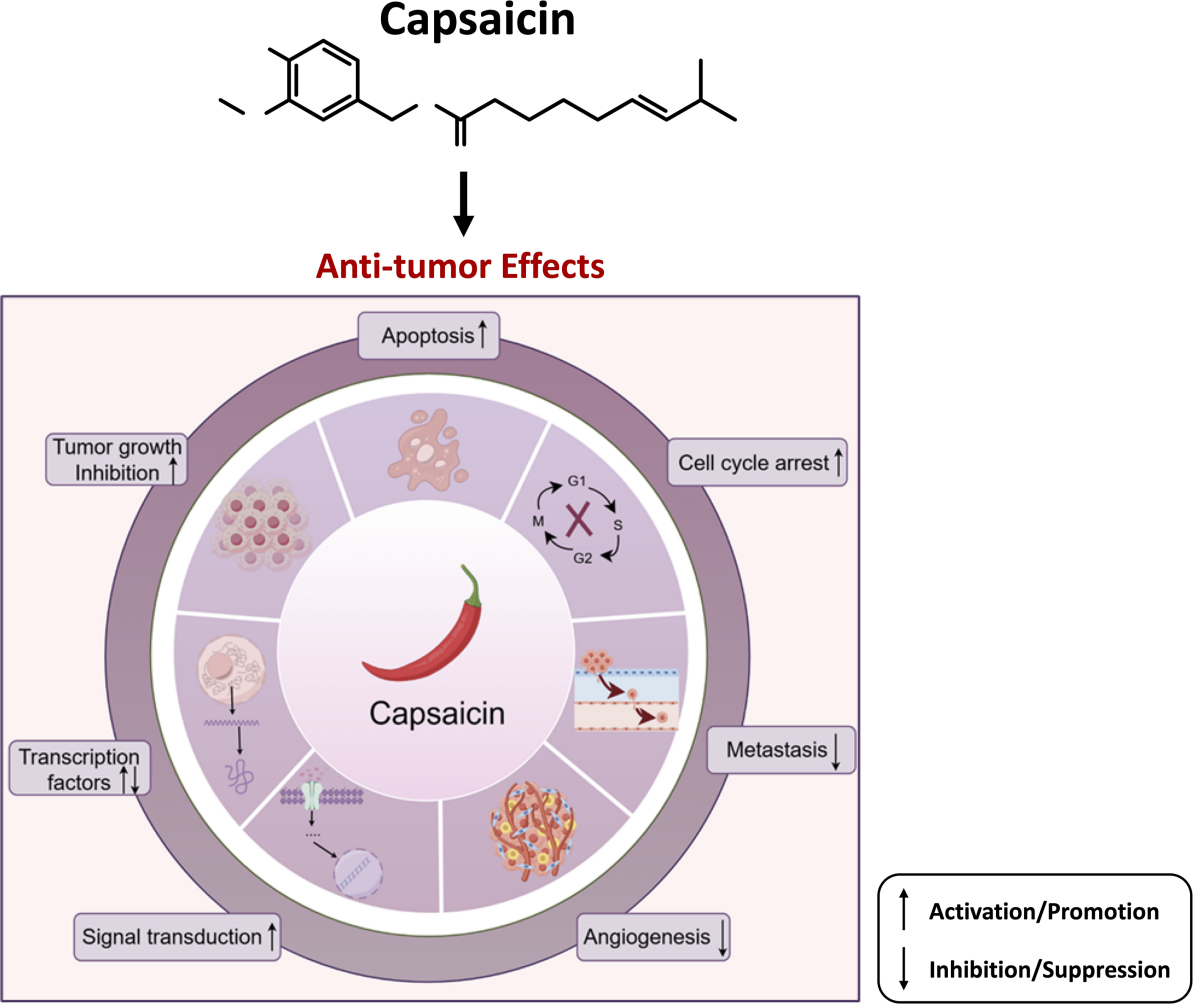
Schematic illustration of capsaicin-mediated antitumor effects. Capsaicin exerts antitumor effects by modulating tumor-intrinsic processes, including apoptosis, cell-cycle arrest, metastasis, angiogenesis, transcription-factor signaling, and signal transduction pathways.

**Figure 2:**
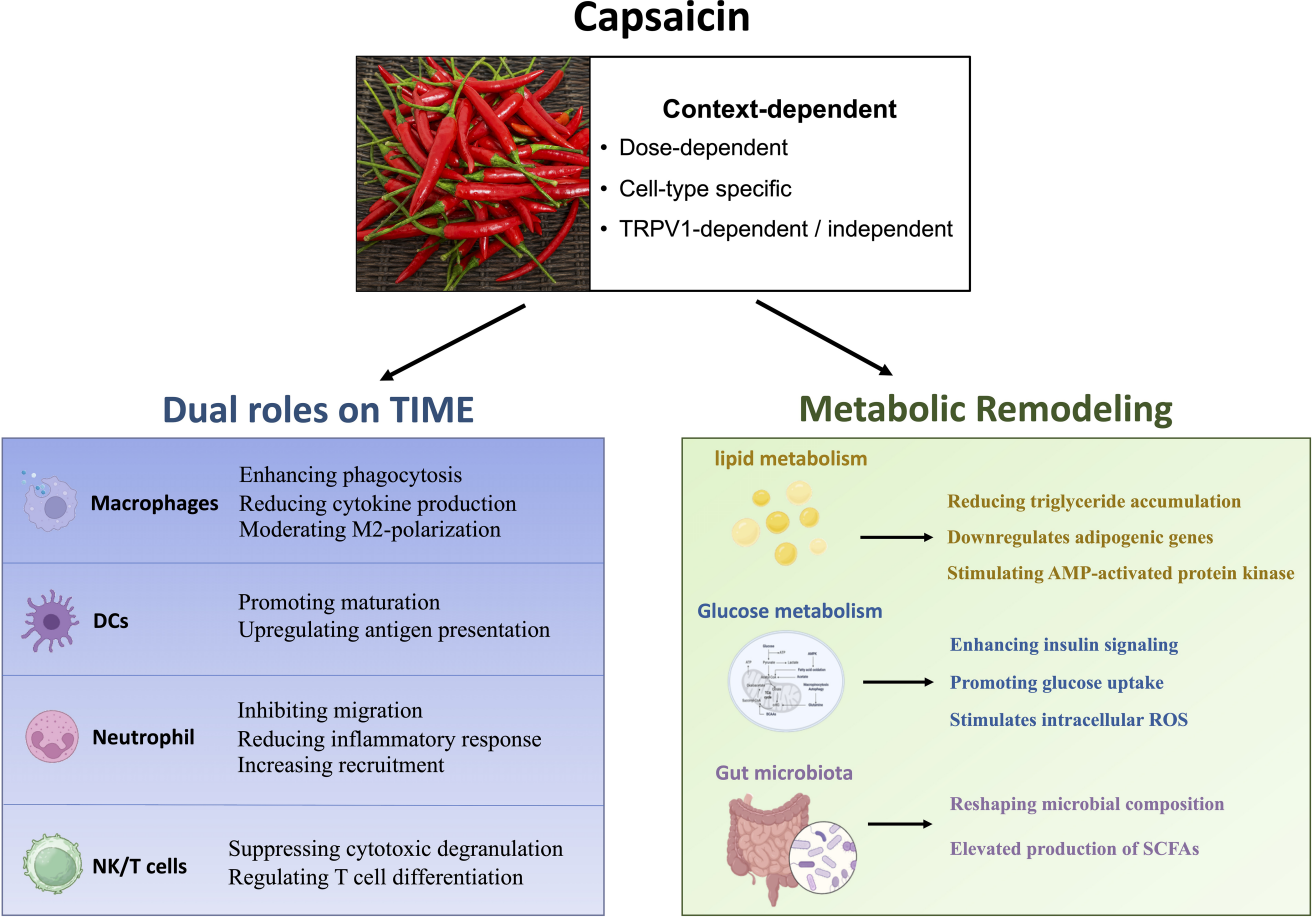
Schematic illustration of capsaicin-mediated immunometabolic regulation. Capsaicin modulates the tumor immune microenvironment and metabolic remodeling through coordinated, context-dependent regulation of immune-cell function, metabolic pathways and gut microbiota composition.

Capsaicin has demonstrated remarkable antitumor potential by acting on multiple molecular and cellular targets. It may exert its effects through the coordinated modulation of tumor biology (**Figure 1**), as well as remodelling TIME and metabolic homeostasis (**Figure 2**)—thereby forming an integrated network of antitumor mechanisms. Moreover, its capacity to elicit complex immunometabolism responses provides new insights into the dynamic interplay between tumour, immune, and metabolic systems.

Additional biological and experimental variables should be considered when interpreting the current evidence base. Although sexual dimorphism has been reported in both TRPV1 signaling and immune regulation, most studies discussed in this review did not specify the sex of experimental models or were not designed to assess sex-specific effects. Consequently, whether capsaicin exerts differential immunological or immunometabolic effects in males and females remains largely undefined and warrants systematic investigation. In parallel, the temporal dynamics of capsaicin responses remain insufficiently characterized. Available evidence suggests that early events, such as Ca²^+^ influx and acute metabolic perturbations, can occur rapidly following TRPV1 activation, whereas downstream immune modulation and metabolic reprogramming are likely to evolve over longer time scales. However, as many studies rely primarily on endpoint measurements, it remains unclear which effects are transient versus sustained.

To address these limitations, future studies should incorporate sex-balanced experimental cohorts and report sex-stratified analyses rather than pooling male and female subjects without adjustment. Given potential sex-dependent differences in TRPV1 expression, nociceptive sensitivity, inflammatory tone, and immune-metabolic responses, dose selection and treatment schedules should be optimized separately in male and female preclinical models when feasible. Moreover, kinetic study designs should include early timepoints, such as minutes to several hours after capsaicin exposure, to capture acute TRPV1-dependent Ca²^+^ signaling and metabolic perturbations; intermediate timepoints, such as 24–72 h, to evaluate cytokine production, antigen presentation, and innate immune activation; and later timepoints over days to weeks to assess sustained remodeling of the tumor immune microenvironment, adaptive immune responses, and antitumor efficacy.

Several important challenges remain. The effects of capsaicin are highly context- and dose-dependent, and the mechanisms governing this functional heterogeneity are not yet fully understood. In addition, most current evidence is derived from *in vitro* or preclinical models, with limited validation in clinically relevant settings. Future studies should prioritize defining context-specific therapeutic windows, resolving cell type– and metabolism-dependent effects, and incorporating longitudinal and sex-stratified designs *in vivo* and in clinical cohorts. Addressing these challenges will be critical for translating capsaicin-based strategies into effective cancer therapies.
